# Development of outcomes for evaluating emergency care triage: a Delphi approach

**DOI:** 10.1186/s13049-023-01073-1

**Published:** 2023-02-25

**Authors:** André Johansson, Anna Ekwall, Jakob Lundager Forberg, Ulf Ekelund

**Affiliations:** 1 Department of Health Sciences, Faculty of Medicine, Lund University, Box 157, 221 00 Lund, Sweden; 2 Department of Emergency Medicine, Helsingborg Hospital, Helsingborg, Sweden; 3 Emergency Medicine, Department of Clinical Sciences Lund, Lund University, Skane University Hospital, Lund, Sweden

**Keywords:** Triage, Outcome measures, Outcome set, Emergency care, Risk assessment, Reference standard

## Abstract

**Background:**

Triage is used as standard of care for prioritization and identification of time-critical patients in the emergency department (ED) globally, but it is unclear what outcomes should be used to evaluate triage. Currently used outcomes do not include important time-critical diagnoses and conditions.

**Method:**

We used 18 Swedish triage experts to collect and assess outcomes for the evaluation of 5-level triage systems. The experts suggested 68 outcomes which were then tested through a modified Delphi approach in three rounds. The outcomes aimed to identify correctly prioritized red patients (in need of a resuscitation team), and orange patients (other time critical conditions). Consensus was pre-defined as 70% dichotomized (positive/negative) concordance.

**Results:**

Diagnoses, interventions, mortality, level of care and lab results were included in the outcomes. Positive consensus was reached for 49 outcomes and negative consensus for 7 outcomes, with an 83% response rate. The five most approved outcomes were the interventions *Percutaneous coronary intervention*, *Surgical airway* and *Massive transfusion* together with the diagnoses *Tension pneumothorax* and *Intracerebral hemorrhage* that received specific interventions. The outcomes with the clearest disapproval included *Admittance to a ward*, *Treatment with antihistamines* and *The ordering of a head computed tomography scan.* The outcomes were considered valid only if occurring in or from the ED.

**Conclusion:**

This study proposes a standard of 49 outcomes divided into two sets tied to red and orange priority respectively, to be used when evaluating 5-level priority triage systems; Lund Outcome Set for Evaluation of Triage (LOSET). The proposed outcomes include diagnoses, interventions and laboratory results. Before widespread implementation of LOSET, prospective testing is needed, preferably at multiple sites.

## Background

The use of triage in the emergency department [ED] is generally motivated by the need of identifying and treating patients with time-critical conditions [1] and by patient safety concerns stemming from waiting times [2]. However, no clear consensus exists for what outcomes to use when evaluating ED triage [3]. Reduction of short-term mortality has been described as a primary reason for triage by both designers of triage systems [4] and their users [5], but a review [3] noted that only 12 out of 66 triage validation studies used *mortality* as a reference outcome. Other outcomes that have been evaluated include *death in the ED*, *admittance to the intensive care unit [ICU]* or to a *ward* [3], *need for an emergency procedure,* and *specific lactate levels* [6].

Another problem is that many triage validation studies are based on outcomes that do not include the identification of time-critical conditions. Missing a time-critical condition (e.g. *anaphylactic shock)* will thereby not be considered a failure if *death* and/or *ICU admission* were avoided due to timely treatment. [3].

Previous validation studies for adults have considered triage to be a one-dimensional problem where all patients with untoward outcomes ideally should be categorized to the highest triage category [red priority] [3]. This is neither reasonable nor consistent with how triage systems intentionally assign some time-critical conditions that do not need a full resuscitation team to the second highest priority [orange priority], such as suspected *testicular torsion* [7, 8]. However outside of pediatric triage [9], the two highest priorities have not been evaluated separately.

The aim of this study was to develop a set of outcomes that could be used for evaluating 5-level ED priority triage systems.

## Methods

### Design

A modified Delphi approach [10] was used. The Delphi method is an iterative process of repeated questionnaires that are bundled with the results of the previous rounds to a panel of experts with the goal of finding consensus. The items to be assessed in the Delphi rounds were gathered in initial interviews with the experts in round one, and from the published literature. The Delphi approach has successfully been used when seeking consensus in emergency care [11], and when assessing questions related to triage [12]

### Panel of experts

To create the expert panel, three important groups were identified: Clinicians working in emergency care, researchers publishing studies related to triage, and designers of ED priority triage systems. To the clinical part of the expert group, we recruited Swedish physicians and registered nurses with a specific interest and knowledge of triage. To the research group, we recruited individuals who were listed as either the first, second or last author of a published study during the last five years related to emergency priority triage and outcomes for adult patients. Lastly, in the designer group we recruited designers or editors of emergency care priority triage systems and persons medically responsible or integral in the implementation of such systems. Representatives from all major triage systems in use in Sweden (RETTS, SATS, WEST) were recruited. The experts in all three groups were recruited from geographically diverse locations in Sweden. If an expert missed any of the questionnaire rounds, they were not excluded from joining the following rounds (Table 1).

**Table 1:** Expert panel demographics

Total included experts	18
Gender	
Male	9
Female	9
Occupation	
Nurse	11
Physician	7
Groups	
Clinicians	10
Researchers	4
Triage systems designers	4
Years of experience in field	
1–5	3
6–9	1
10–15	4
16 +	10
Average	14

### Data collection

The data collection was split into collecting suggested outcomes (round one) during three weeks in the spring of 2021, and testing of these outcomes by a Delphi approach (round two to four) during the last months of 2021. The experts were informed that the term outcome could include any measurable outcome that they felt could be relevant to evaluate triage, including e.g. admission to in-hospital care, diagnosis, interventions or laboratory testing.

#### Round one: collection of outcome proposals and creation of Delphi questionnaire

The outcomes to be assessed were gathered through qualitative semi-structured interviews using written notes shared with the expert. During the interview, the experts suggested outcomes and assigned them to either of two groups: Outcomes that would motivate red priority at the ED, i.e. with resuscitation team activation, and outcomes with no need for resuscitation team activation but that are still considered time-critical; i.e. that would motivate red or orange priority. After round one the experts could not suggest new outcomes.

**Fig. 1:**
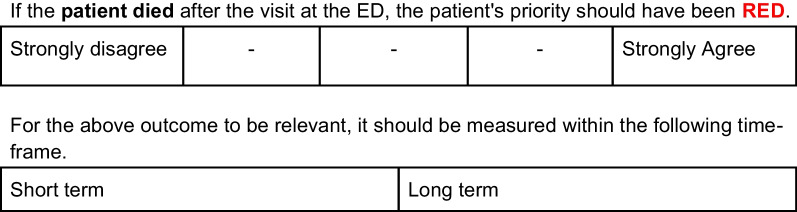
Outcome question without a specified time-frame, followed by a question regarding time-frame

**Fig. 2:**
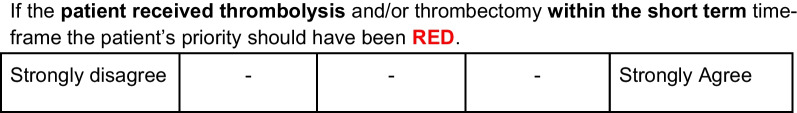
Outcome question with the time-frame included in the definition of the outcome

For every proposed outcome the experts were asked to also suggest a time-frame within which the outcome should be evaluated. When the suggested time-frames for the outcome varied among the experts, a question of the time-frame was included separately in the Delphi questionnaire, see Fig. 1. If only similar time-frames were suggested, the time-frame was embedded in the outcome description, exemplified in Fig. 2. Two groups of time-frames emerged from the interviews; *short-term*, often described as”in the ED'' or up to a day from leaving the ED, and *long-term*, described as a couple of days up to 30 days. Since no clear cutoffs were presented in the interviews, we included this as a question at the beginning of the questionnaire, intentionally leaving the cut offs as overlapping, see Table 2.

**Table 2:** Suggested time-frames

Short term	Long term
In the ED < reached consensus >	Within 12 h from leaving the ED
Within 2 h from leaving the ED	Within 24 h from leaving the ED
Within 6 h from leaving the ED	Within 3 days from leaving the ED
Within 12 h from leaving the ED	Within 7 days from leaving the ED
Within 24 h from leaving the ED	Within 30 days from leaving the ED

At the end of each interview the suggested outcomes were repeated back to the experts to confirm that they were understood correctly. The interviews were conducted during the spring of 2021 and held through video calls due to the COVID-19 pandemic. Two pilot interviews were conducted with emergency care specialist nurses before the first interview, which led to changes in how the questions were presented, to avoid misunderstandings.

The Delphi questionnaire was constructed and answered through REDCap which is a web-based platform that provides secure, web-based access to research data and tools to gather it through questionnaires [13, 14]. Our questionnaire was written in Swedish and piloted on the above mentioned specialist nurses before being sent to the experts.

#### Rounds two to four

Subsequent rounds followed a modified Delphi process as described by Clayton [10]. Experts were invited to the Delphi questionnaire via individual emails sent through REDCap. The outcomes gathered in round one were presented to the experts together with the experts' arguments for or against the outcomes. Potential for conflict between different outcomes were described next to the affected outcome, such as that positive consensus of one outcome could make another redundant. *Admission to ward* was added as an outcome in round two based on previous research; the inclusion of this outcome was planned beforehand. The experts were informed that outcomes could be added by the researchers, but not of which specific outcomes.

All outcomes were presented as statements with a similar structure including both outcome and priority level as seen in Figs. 1 and 2. The outcomes were assessed via a five-stepped Likert scale from “Strongly agree” to “Strongly disagree”. The experts also had the possibility, through a free text input, to supplement their opinion with new arguments that they believed were missing. These arguments were analyzed with manifest qualitative content analysis [15] and arguments for or against outcomes were presented verbatim alongside the outcomes in the following rounds. From round three, the aggregated expert opinions of the previous rounds were displayed alongside the outcome, and from round four this included stability. All questionnaires were sent out with three reminders and the experts had one month to answer each questionnaire.

### Data analysis

The data from the Delphi questionnaires were extracted from REDCap and analyzed in Microsoft Excel 2013 for Windows. The predetermined cutoff for consensus was that 70% of the responses fell in either of the upper two alternatives of the five-step Likert scale [16], i.e. positive consensus, or in the lower two alternatives, i.e. negative consensus. Stability was similarly deemed reached if 70% of the answers did not change from its dichotomized group in the previous round, i.e. positive or negative. If an outcome reached the predetermined cutoffs for consensus and stability, it was excluded from further rounds. This could happen at earliest after round three since stability required two rounds of questionnaires to be calculated. For the time-frames, consensus/stability was calculated per suggested cutoff in each group (long term/short term).

Since all outcomes that gained consensus also reached stability it was decided to present median and interquartile range [IQR] for all outcomes based on measures of spread [17]. The round where consensus was reached was recorded together with the median and IQR from the round when both consensus and stability were reached.

## Results

In total, 18 experts were recruited, 10 in the clinical group and four each in the researcher and design groups. The experts in the research and designer groups all had a clinical background in emergency medicine and care, and most of them were still partly clinically active. The experts had worked an average 14 years with ED triage. All of the groups had an even spread between genders (50%/50%) and profession (61% registered nurses, 39% physicians). The total response rate in all rounds was 83%, and the rounds are described in Table 3.

**Table 3:** Response rate based on expert group

	Round one (%)	Round two (%)	Round three (%)	Round four (%)	Total all rounds (%)
Clinicians	10/10	9/10	6/10	6/10	21/30
Researchers	4/4	4/4	2/4	4/4	10/12
Designers	4/4	4/4	4/4	3/4	11/12
Total	18/18 (100%)	17/18 (94%)	12/18 (66%)	13/18 (72%)	60/72 (83%)

In round one the experts suggested 67 outcomes, and one (*admission to ward)* was added from previous research, yielding 68 outcomes to be assessed in the Delphi rounds. In these, there was positive consensus for 49 outcomes, i.e. approval of the outcome, and negative consensus for seven outcomes, i.e. disapproval, as described in Table 4. This was split between red priority where 38 outcomes reached positive consensus and two reached negative consensus, and orange priority where eleven/five reached positive/negative consensus. The outcome *admission to ward* reached negative consensus. Some outcomes that were suggested for both red and orange priority reached consensus for both priorities, and some outcomes that were similarly formulated also reached consensus. These outcomes are marked with roman numerals in the “Conflict” column in Table 4, and are further clarified below the table.

**Table 4:** Results from Delphi rounds—outcomes that reached consensus

Outcome—highest/red priority	Median	IQR	Consensus in round	Pos/neg consensus	Conflict
Patient received PCI within the short term time-frame	5	0	1	Pos	
Patient received a surgical airway within the short term time-frame	5	0	1	Pos	
Patient received massive transfusion within the short term time-frame	5	0	1	Pos	
Diagnosis: intracerebral hemorrhage and patient was admitted to the ICU or received interventional care for the hemorrhage	5	0	1	Pos	
Diagnosis: tension pneumothorax	5	0	1	Pos	
Patient received thrombolysis and/or thrombectomy within the short term time-frame	5	0.25	1	Pos	
Patient was intubated, received an oropharyngeal/nasal airway or laryngeal mask within the short term time-frame	5	0.25	1	Pos	
Patient received a tourniquet during the visit in the ED	5	0.25	1	Pos	
Diagnosis: anaphylaxis and patient received adrenaline	5	0.25	1	Pos	I
Diagnosis: STEMI	5	0.25	1	Pos	II
Patient received mechanical ventilation	5	1	1	Pos	
Patient received a glucagon injection due to hypoglycemia during the ED visit	5	1	1	Pos	
Patient received inotropic support during the ED visit	5	1	1	Pos	
Patient's hemoglobin was under 60 g/L and the patient had an ongoing bleed	5	1	1	Pos	
Patient's pO2 was under 4.5 kPa measured arterially within the short term time-frame	5	1	1	Pos	
Diagnosis: bacterial meningitis	5	1	1	Pos	
Diagnosis: ventricular tachycardia	5	1	1	Pos	
Diagnosis: anaphylaxis	4.5	1	1	Pos	I
Diagnosis: addisonian crisis	4.5	1	1	Pos	
Diagnosis: pulmonary edema	4.5	1	1	Pos	
Patient died at or after seeking care at the ED	4	0	2	Pos	
Patient admitted to ICU	4	0	1	Pos	
Patient admitted to step down unit (Intermediate Care Unit)	4	0	3	Pos	III
Patient was operated on or received a surgical intervention within an operation room within the short term time-frame	4	0	1	Pos	IV/V
Patient was planned/booked for immediate surgery within the short term time-frame	4	0	1	Pos	IV/V
Patient received glucose due to hypoglycemia during the ED visit	4	0	1	Pos	
Patient received an antidote directly related to the chief complaint during the ED visit	4	0	1	Pos	
Diagnosis: Perforated ulcer	4	0	1	Pos	
Diagnosis: Third degree (complete) heart block	4	0	1	Pos	
Patient received high flow oxygen (> 30L/min)	4	0.25	1	Pos	
Patient's pCO2 was over 9 kPa within the short term time-frame	4	0.25	1	Pos	
Patient's pH was under 7.3 within the short term time-frame	4	0.25	2	Pos	
Diagnosis: Sepsis and NEWS2 score of > 6p at first assessment after being admitted	4	0.25	2	Pos	VI
Patient's lactate levels were over 5 mmol/L within the short term time-frame	4	0.5	2	Pos	
Patient received a pacemaker or was put on external pacing within the short term time-frame	4	1	1	Pos	
Diagnosis: Ectopic pregnancy	4	1	1	Pos	
Any diagnosis related to internal traumatic injuries of organs in abdomen, thorax or head	4	1	1	Pos	
Diagnosis: Sepsis	4	1.25	1	Pos	VI
Patient's creatinine was doubled compared to previous values	2	1	2	Neg	
Patient admitted to ward	1	0	1	Neg	

Second highest/orange priority	Median	IQR	Consensus in round	Pos/neg consensus	Conflict
Diagnosis: testicular torsion	5	0.25	1	Pos	V
Diagnosis: ovarian torsion	5	0.25	1	Pos	V
Patient was operated on or received a surgical intervention within an operation room within the short term time-frame	4	0	1	Pos	IV
Diagnosis: Sepsis and NEWS2 score of < 7p at first check when admitted	4	0	3	Pos	VI
Diagnosis: STEMI	4	0	1	Pos	II
Patient admitted to step down unit (Intermediate Care Unit)	4	0.25	1	Pos	III
Diagnosis: NSTEMI or unstable angina	4	0.25	1	Pos	
Patient received NIV/BiPap/CPap in the ED or after	4	1	1	Pos	
Any diagnosis of an open fracture	4	1	1	Pos	
Diagnosis: femur fracture	4	1	2	Pos	
Any diagnosis related to acute ischemia	4	1	1	Pos	
Diagnosis: pulmonary embolism	2	0	3	Neg	
Patient received Nitroglycerine in the ED	2	0.25	2	Neg	
Patient admitted to the acute care ward	2	0.5	2	Neg	
Patient received antihistamines in the ED	1.5	1	1	Neg	
A head CT scan was ordered on the patient in the ED	1	0.75	2	Neg	

All outcomes that reached positive consensus and stability also reached consensus and stability regarding that they should be evaluated within the short-term time-frame, which in itself reached consensus for the alternative *In the ED*, see Table 2. This means that the outcomes should only be counted if they occurred in the ED or in some cases, initiated directly from the ED in the case of hospital admission or immediate surgery.

(I) Two variations of how anaphylaxis should be evaluated reached positive consensus for red priority.
(II) STEMI reached consensus for both red and orange priority.
(III) Admittance to a step-down unit (Intermediate Care Unit) reached positive consensus for both red and orange priority.
(IV) Two different outcomes to measure the occurrence of immediate/early surgical intervention reached positive consensus for red priority and one for orange.
(V) Two diagnoses that lead to immediate/early surgical intervention reached positive consensus for orange priority, see also conflict IV.
(VI) Two variations of how sepsis should be evaluated reached positive consensus for red priority, and one reached positive consensus for orange priority.

## Discussion

The main result of this study is the proposal of 49 outcomes grouped in two priority-specific sets to be used for the validation of ED triage systems for adults, as well as the recommendation against seven outcomes. We have chosen to call the outcome groups Lund Outcome Set for Evaluation of Triage (LOSET).

### Comparison to other comprehensive sets of outcomes

To our knowledge the only other comprehensive work on outcomes in the evaluation of triage systems has been done by van Veen et al. [9] in pediatric patients. In this study, the authors evaluated a specific triage system (SATS) for a specific study, with the methodology focused on the evaluation at hand and not the development of the outcomes. In contrast, LOSET was developed independently of any specific study with a methodology focused on the selection of outcomes, and is by design triage system agnostic. Further, van Veen et al. evaluated the highest priority based only on vital parameters, and we argue that this creates the possibility of circular logic that LOSET does not have: A triage system where the cutoffs for vital signs are based on the reference values for red priority will in principle never be wrong. Moreover, diagnoses such as sepsis or meningitis were found In the second highest priority in van Veens work, but in the highest priority in the present study. In a subsequent sudy, Hansen et al. [18] combined the highest two priorities in van Veen’s study to validate the highest priority in a pediatric population, making the third highest priority comparable to the second highest priority (orange) in our study. This would increase the disparity even further, with no common outcomes except one that was disapproved in this study; *A head CT scan was ordered on the patient in the ED*.

### Singular outcomes used in other studies

Previous studies evaluating triage for adults have used some of LOSET’s proposed outcomes: *mortality, admittance to the ICU, acute surgical intervention* and *lactate over 5 mmol/L* [3, 6]. *Admittance to a ward,* which was the outcome with the clearest negative consensus in this study (median 1,00, IQR 0,00), has also been used. Spangler [19] used *hospital admission* as an outcome because of “face validity”, perhaps also because of its previous use in multiple studies [20–22]. Its inversion, *discharged from the ED*, has been used to indicate low acuity [3]. The negative consensus for *admission to a ward* in the present study could probably be explained by the fact that not all admissions are time-critical, and that the experts valued triage specificity.

### Conflicts in LOSET

There were six identified conflicts in the results. These could be viewed from a methodological perspective; can a Delphi approach efficiently reach a dichotomous consensus if an outcome is proposed in many forms, or is it more methodologically sound to first reach a consensus on which version of the outcome to use? An alternative is that the complexity of the research question might make it difficult to reach a dichotomized consensus. One conflict (VI) arose from a proposal to supplement a sepsis diagnosis with vital signs assessed at ward admission (which avoids circular logic, as the vital signs at triage are not used, see above). Including vital signs in the sepsis outcome could also lessen the effect on triage specificity that liberally made sepsis diagnoses could create. The apparent risk, however, is that early treatment in the ED will reverse or stop a septic patient from developing shock, and that effective treatment could thereby make a red priority “wrong”. Testicular or ovarian torsion (conflict V) were assigned to orange priority by our experts to save resources, i.e. to not activate a resuscitation team that red priority generally leads to. However, by including *acute surgical intervention* in the red priority, both these conditions could be correctly evaluated as red. All of the conflicts above could be viewed as stemming from the will to limit overtriage, which seems like a recurring theme in the results.

### Application of LOSET

We suggest assessing both red and orange priority as correct in conflicts II-VI. For conflict I, we would suggest disregarding adrenaline use in anaphylaxis since this outcome should be easier to use. Although developed for ED triage, LOSET should also be possible to use in the evaluation of pre-hospital priority triage, including ambulance dispatch telephone triage, since the goal in these situations are generally the same as in the ED, i.e. identifying time critical conditions. The outcome set could also form a basis for the evaluation of care level triage which is often applied in the form of telephone triage, where the goal is to direct the patient to the optimal level of care, such as the ED, urgent care, primary care etc. However, it should be noted that LOSET is focused on time-critical conditions, which is likely only a subset of all conditions that that should be referred to the ED.

## Strengths and limitations

Even if the research question fits the Delphi methodology, i.e. generation of consensus on complex questions [23], the conflicts in the results indicate a methodological problem. However, most outcomes proposed do not include conflicts, and it seems reasonable to assume that multiple studies are needed to optimize the LOSET outcomes. Followup studies with other methodologies may answer some of the questions raised by the present results.

This study included a panel with expertise evident through their experience with triage, their diverse and relevant backgrounds, and the richness of suggestions for outcomes. There is no clear consensus on the panel size in Delphi studies, but our size of 18 is in accordance with Clayton [10] who recommends groups of 15–30 panelists if the group is heterogeneous such as ours. The fact that all experts were from Sweden could limit the results’ transferability to other countries. Transferability could also be a problem towards triage systems without five levels, and in pediatric systems. However we find it likely that any system that considers the top two priorities as time-critical and uses the highest priority to call on a resuscitation team can apply most, if not all, of the outcomes suggested in the present study.

## Conclusion

This study proposes a standard of 49 outcomes divided into two sets tied to red and orange priority respectively, to be used when evaluating 5-level priority triage systems; Lund Outcome Set for Evaluation of Triage (LOSET). The proposed outcomes include diagnoses, interventions and laboratory results. Before widespread implementation of LOSET, prospective testing is needed, preferably at multiple sites.

## Data Availability

The datasets used and/or analyzed during the current study are available from the corresponding author on reasonable request.

## Abbreviations

BiPap: Bilevel positive airway pressure
CT: Computed tomography
CPap: Continuous positive airway pressure
ED: Emergency department
ICU: Intensive care unit
IQR: Interquartile range
LOSET: Lund Outcome Set for Evaluation of Triage
NEWS2: National Early Warning Score
NIV: Non-invasive ventilation
PCI: Percutaneous coronary intervention
RETTS: Rapid Emergency Triage and Treatment System
SATS: South African Triage Scale
SBU: Statens beredning för medicinsk och social utvärdering
STEMI: ST Elevation Myocardial Infarction
WEST: WEst coast System for Triage

## References

[1] Farrokhnia N, Göransson KE (2011). Swedish emergency department triage and interventions for improved patient flows: a national update. Scand J Trauma Resusc Emerg Med.

[2] Wireklint SC, Elmqvist C, Parenti N, Göransson KE (2018). A descriptive study of registered nurses’ application of the triage scale RETTS©; a Swedish reliability study. Int Emerg Nurs.

[3] Zachariasse JM, van der Hagen V, Seiger N, Mackway-Jones K, van Veen M, Moll HA (2019). Performance of triage systems in emergency care: a systematic review and meta-analysis. BMJ Open.

[4] Widgren, B. (2012). RETTS: *Akutsjukvård direkt*. Studentlitteratur.

[5] Statens beredning för medicinsk och social utvärdering. (2010). *Triage och flödesprocesser på akutmottagningen: En systematisk litteraturöversikt* (SBU-rapport, 197). Stockholm: SBU

[6] Levin S, Toerper M, Hamrock E, Hinson JS, Barnes S, Gardner H, Douglas A, Linton B, Kirsch T, Kelen G (2018). Machine-learning-based electronic triage more accurately differentiates patients with respect to clinical outcomes compared with the Emergency Severity Index. Ann Emerg Med.

[7] Gilboy, N., Tanabe, P., Travers, D., & Rosenau, A. M. (2020) *Implementation handbook 2020 edition: ESI Emergency Severity Index.* (Version 4). Emergency Nurses Association

[8] Predicare. (2022). RETTS-online (Version 2022) [Web-app]. Predicare. https://rettsonline-app.com

[9] van Veen M, Steyerberg EW, Ruige M, van Meurs AHJ, Roukema J, van der Lei J, Moll HA (2008). Manchester triage system in pediatric emergency care: prospective observational study. BMJ.

[10] Clayton MJ (2006). Delphi: a technique to harness expert opinion for critical decision-making tasks in education. Educ Psychol.

[11] Wihlborg J, Edgren G, Johansson A, Sivberg B (2014). The desired competence of the Swedish ambulance nurse according to the professionals—a Delphi study. Int Emerg Nurs.

[12] Khorram-Manesh A, Burkle FM, Nordling J, Goniewicz K, Faccincani R, Magnusson C, Merzaai B, Ratnayake A, Carlström E (2022). Developing a translational triage research tool: part two—evaluating the tool through a Delphi study among experts. Scand J Trauma Resusc Emerg Med.

[13] Harris PA, Taylor R, Thielke R, Payne J, Gonzalez N, Conde JG (2009). Research electronic data capture (REDCap)—a metadata-driven methodology and workflow process for providing translational research informatics support. J Biomed Inform.

[14] Harris PA, Taylor R, Minor BL, Elliott V, Fernandez M, O’Neal L, McLeod L, Delacqua G, Delacqua F, Kirby J, Duda SN (2019). The REDCap consortium: Building an international community of software platform partners. J Biomed Inform.

[15] Lundman, B., & Hällgren Graneheim, U. (2017). Kvalitativ innehållsanalys. B. Höglund-Nielsen & M. Graneskär (Red). *Tillämpad kvalitativ forskning inom hälso- och sjukvård.* (Tredje upplagan. s. 219–233). Studentlitteratur.

[16] Hsu C-C, Sandford BA (2007). The Delphi technique: making sense of consensus. Pract Assess Res Eval.

[17] Varndell W, Fry M, Lutze M, Elliott D (2020). Use of the Delphi method to generate guidance in emergency nursing practice: a systematic review. Int Emerg Nurs.

[18] Hansen LH, Mogensen CB, Wittenhoff L, Skjöt-Arkil H (2017). The danish regions pediatric triage model has a limited ability to detect both critically ill children as well as children to be sent home without treatment—a study of diagnostic accuracy. Scand J Trauma Resusc Emerg Med.

[19] Spangler D, Hermansson T, Smekal D, Blomberg H (2019). A validation of machine learning-based risk scores in the prehospital setting. PLoS ONE.

[20] Aeimchanbanjong K (2017). Validation of different pediatric triage systems in the emergency department. World J Emerg Med.

[21] Gravel J, Fitzpatrick E, Gouin S, Millar K, Curtis S, Joubert G, Boutis K, Guimont C, Goldman RD, Dubrovsky AS, Porter R, Beer D, Doan Q, Osmond MH (2013). Performance of the Canadian triage and acuity scale for children: a multicenter database study. Ann Emerg Med.

[22] Gräff I, Goldschmidt B, Glien P, Bogdanow M, Fimmers R, Hoeft A, Kim S-C, Grigutsch D (2014). The German Version of the Manchester Triage System and its quality criteria—first assessment of validity and reliability. PLoS ONE.

[23] Powell C. The Delphi technique: myths and realities. J Adv Nursing. 2013;41(4):376–82. 10.1046/j.1365-2648.2003.02537.x. 10.1046/j.1365-2648.2003.02537.x 12581103

